# Myocardial Infarction With Non-obstructive Coronary Arteries (MINOCA) Revealing Coronary Embolism From Left Atrial Appendage Thrombus in Rheumatic Valvular Atrial Fibrillation

**DOI:** 10.7759/cureus.110659

**Published:** 2026-06-11

**Authors:** Mehdi Moujahid, Hafsa Erregui, Hicham Faliouni, Zouhair Lakhal, Aatif Benyass

**Affiliations:** 1 Cardiology, Mohammed V Military Teaching Hospital, Rabat, MAR

**Keywords:** atrial fibrillation, coronary embolism, left atrial appendage thrombus, minoca, rheumatic heart disease

## Abstract

Myocardial infarction with non-obstructive coronary arteries (MINOCA) represents a diagnostic challenge with multiple underlying mechanisms, including coronary embolism. We report the case of a 50-year-old woman with a history of rheumatic fever who was admitted for acute chest pain. Electrocardiography showed atrial fibrillation with inferolateral ischemic changes, and elevated troponin levels confirmed myocardial infarction. Transthoracic echocardiography revealed moderate mitral stenosis, severe aortic stenosis, and left atrial enlargement. Coronary angiography demonstrated thrombotic occlusion of the second obtuse marginal branch without significant atherosclerotic disease. Transesophageal echocardiography identified a left atrial appendage thrombus, supporting a cardioembolic mechanism. This case emphasizes the importance of considering coronary embolism in MINOCA, particularly in patients with valvular atrial fibrillation, and highlights the role of a structured diagnostic approach in guiding diagnosis and management.

## Introduction

Acute myocardial infarction is classically related to rupture or erosion of an atherosclerotic plaque, followed by thrombotic obstruction of a coronary artery. However, some patients meet the clinical and biochemical criteria for myocardial infarction while coronary angiography shows no obstructive coronary artery disease. This presentation is referred to as myocardial infarction with non-obstructive coronary arteries (MINOCA) [1]. Unlike conventional occlusive myocardial infarction, MINOCA should not be regarded as a final diagnosis, but rather as an initial diagnostic framework that requires further investigation to determine the underlying cause.

MINOCA accounts for approximately 5-10% of myocardial infarctions and remains challenging because it includes several distinct mechanisms [1]. These include coronary vasospasm, spontaneous coronary artery dissection, coronary microvascular dysfunction, non-obstructive plaque disruption, and coronary embolism [2]. Coronary embolism is less frequent than other mechanisms but may be overlooked, especially when an embolic source is not actively searched for [3]. Atrial fibrillation and structural heart disease are important clinical clues. In rheumatic valvular disease, particularly mitral stenosis, left atrial enlargement and blood stasis favor thrombus formation, especially within the left atrial appendage, and may lead to systemic or coronary embolization [4].

The diagnosis of coronary embolism can be supported by the Shibata criteria, which combine angiographic findings, the identification of an embolic source or embolic risk factors, systemic embolization, and the exclusion of alternative causes such as plaque rupture or atherosclerotic thrombosis [3]. In the diagnostic workup of MINOCA, cardiac magnetic resonance imaging may help differentiate myocardial infarction from myocarditis or Takotsubo syndrome, while optical coherence tomography (OCT) or intravascular ultrasound (IVUS) can reveal occult plaque disruption or spontaneous coronary artery dissection when feasible. Transesophageal echocardiography is particularly useful when a cardioembolic mechanism is suspected.

We report a case of MINOCA caused by coronary embolism from a left atrial appendage thrombus in a patient with rheumatic valvular atrial fibrillation. This case underlines the need to consider coronary embolism in MINOCA, particularly in patients with atrial fibrillation or valvular heart disease, and highlights the importance of a structured diagnostic approach to guide appropriate management.

## Case presentation

A 50-year-old woman with no conventional cardiovascular risk factors and a history of rheumatic fever, treated with long-term benzathine penicillin prophylaxis, presented three hours after the onset of acute chest pain. On admission, she was hemodynamically stable, with a blood pressure of 127/64 mmHg and a heart rate of 87 beats per minute. Cardiovascular examination revealed an ejection systolic murmur suggestive of aortic stenosis. There were no clinical signs of acute heart failure, peripheral hypoperfusion, or systemic embolization at initial evaluation. Electrocardiography showed atrial fibrillation with ST-segment depression in the inferior and low lateral leads, associated with T-wave inversion in the same territories (Figure 1).

**Figure 1:**
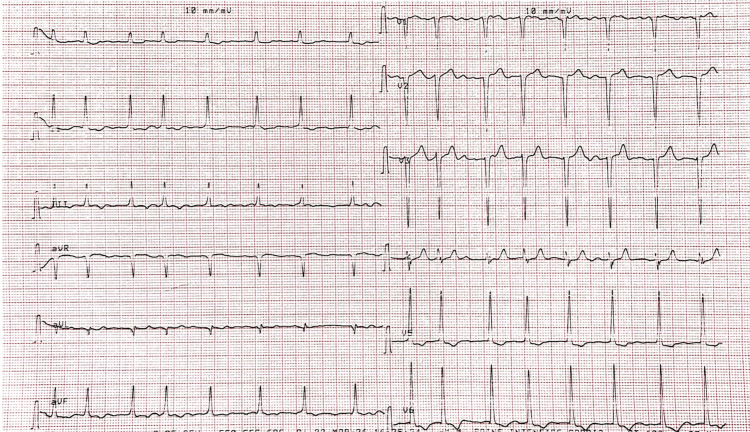
Electrocardiogram on admission showing atrial fibrillation with ST-segment depression in the inferior (II, III, aVF) and lateral leads (V4–V6), associated with T-wave inversion, consistent with myocardial ischemia

Laboratory testing revealed an initial high-sensitivity troponin level of 80 ng/L, rising to 29,000 ng/L, consistent with acute myocardial infarction.

Transthoracic echocardiography demonstrated left ventricular hypertrophy with lateral wall hypokinesia and a preserved ejection fraction of 54%. Severe aortic stenosis was identified, with a mean gradient of 54 mmHg, peak velocity of 4.48 m/s, and valve area of 0.87 cm² (Figure 2). Left atrial enlargement was also noted (Figure 3). Retrospectively, the combination of atrial fibrillation, rheumatic valvular disease, left atrial enlargement, and absence of conventional cardiovascular risk factors represented important clinical clues suggesting a possible embolic mechanism once MINOCA was suspected.

**Figure 2:**
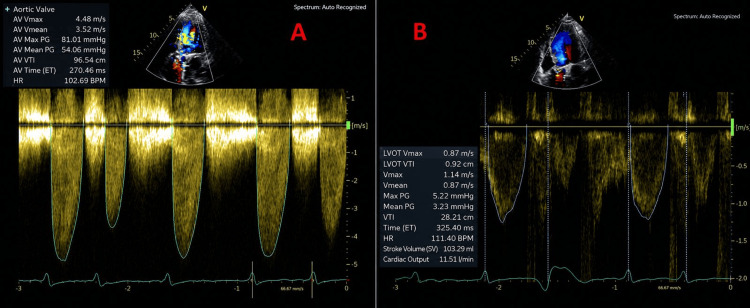
Transthoracic echocardiography demonstrating severe aortic stenosis A) Continuous-wave Doppler showing a peak velocity of 4.48 m/s and a mean gradient of 54 mmHg; B) Aortic valve area estimated at 0.87 cm² using the continuity equation.

**Figure 3:**
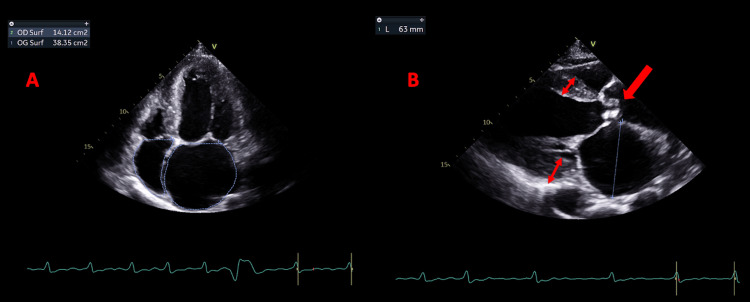
Transthoracic echocardiography findings A) Apical four-chamber view showing left atrial enlargement; B) Parasternal long-axis view demonstrating a calcified aortic valve with restricted opening and left ventricular hypertrophy.

Coronary angiography revealed thrombotic occlusion of the distal obtuse marginal branch, with otherwise normal coronary arteries (Figure 4).

**Figure 4:**
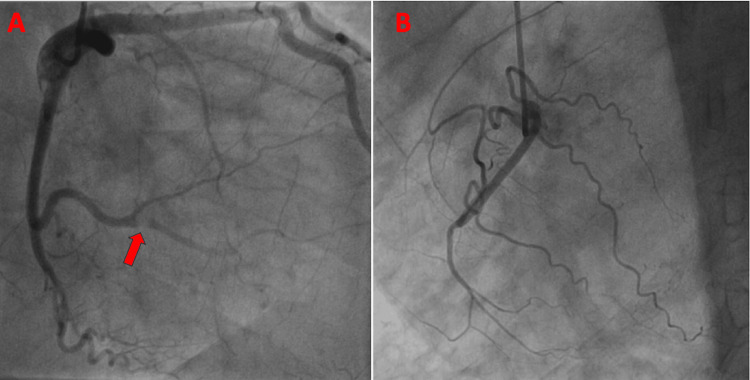
Coronary angiography showing thrombotic occlusion of the distal obtuse marginal branch, with otherwise normal coronary arteries A) Left coronary angiography showing distal thrombotic occlusion of the obtuse marginal branch; the arrow indicates the site of occlusion; B) Right coronary angiography showing no significant atherosclerotic disease.

Given the absence of atherosclerotic disease and the presence of atrial fibrillation, an embolic mechanism was suspected. Transesophageal echocardiography identified a thrombus within the left atrial appendage (Figure 5), confirming a cardioembolic source.

**Figure 5:**
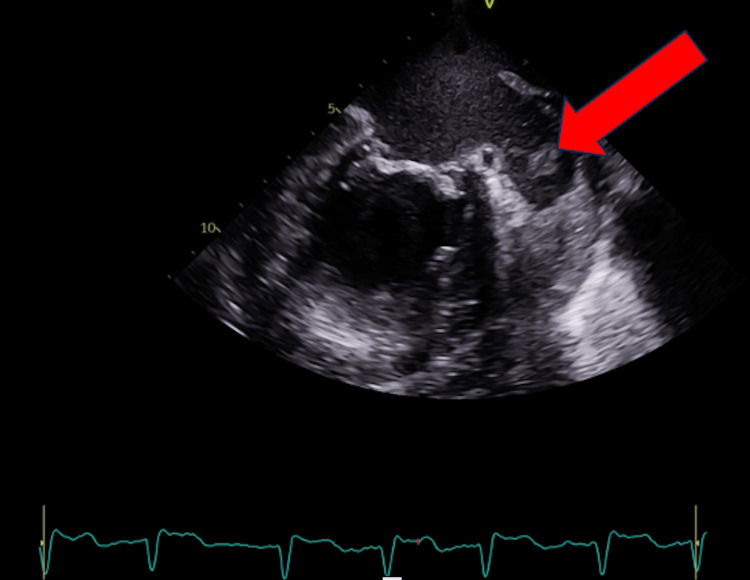
Transesophageal echocardiography showing a thrombus within the left atrial appendage The arrow indicates the left atrial appendage thrombus.

The patient was initially treated with therapeutic anticoagulation using low-molecular-weight heparin (enoxaparin), followed by transition to long-term vitamin K antagonist therapy with regular INR monitoring. Because of rheumatic valvular atrial fibrillation and the presence of a left atrial appendage thrombus, anticoagulation was planned indefinitely, with a target INR of 2.0-3.0. Beta-blocker therapy and guideline-directed medical treatment were also initiated. Given the embolic mechanism and the absence of significant atherosclerotic coronary disease, no coronary intervention was performed. The clinical course was favorable, with no recurrent ischemic events. In view of the severe symptomatic aortic stenosis, the patient was referred for surgical evaluation for aortic valve replacement. A diagnosis of myocardial infarction secondary to coronary embolism was established.

## Discussion

MINOCA represents a heterogeneous working diagnosis requiring a structured diagnostic approach to identify the underlying mechanism and guide management [1]. Failure to determine the etiology may result in inappropriate treatment and an increased risk of recurrence.

Coronary embolism is an infrequent but important cause of MINOCA. In most cases, the embolic source is cardiac, particularly in patients with atrial fibrillation, where blood stasis within the left atrium and its appendage promotes thrombus formation [3]. This risk is further increased in rheumatic mitral stenosis, which contributes to left atrial enlargement and a prothrombotic state [4].

In this case, several findings supported an embolic origin, including abrupt distal coronary occlusion without atherosclerotic disease and the presence of a left atrial appendage thrombus on transesophageal echocardiography. The coexistence of valvular atrial fibrillation further strengthened this mechanism.

Diagnostic criteria for coronary embolism include angiographic features, identification of an embolic source, and exclusion of alternative mechanisms such as plaque rupture [3]. These criteria were fulfilled in this patient, supporting the diagnosis of coronary embolism.

However, cardiac magnetic resonance imaging was not performed; therefore, myocarditis and Takotsubo syndrome were not formally excluded. In addition, intravascular imaging with OCT or IVUS was not performed to rule out occult plaque disruption or spontaneous coronary artery dissection. These points represent limitations of this case, although the overall clinical, angiographic, and echocardiographic findings strongly supported an embolic coronary mechanism.

Transesophageal echocardiography plays a key role by allowing optimal visualization of the left atrial appendage and improved detection of intracardiac thrombi. Its use should be considered when an embolic mechanism is suspected.

From a therapeutic perspective, distinguishing coronary embolism from atherosclerotic myocardial infarction is essential, as long-term management differs. In patients with valvular atrial fibrillation, anticoagulation with vitamin K antagonists remains the recommended strategy, as direct oral anticoagulants are not indicated [5].

In classic occlusive myocardial infarction related to atherosclerotic plaque rupture, early revascularization and antiplatelet therapy are central components of management. In contrast, MINOCA treatment should be individualized according to the underlying mechanism. In the present case, the distal location of the thrombotic occlusion, the absence of significant atherosclerotic disease, and the identification of a left atrial appendage thrombus supported an embolic mechanism. Therefore, no percutaneous coronary intervention (PCI) or thrombectomy was performed, and long-term anticoagulation was considered the cornerstone of management.

This case highlights the importance of considering coronary embolism in MINOCA, particularly in patients with atrial fibrillation and rheumatic valvular disease. Early recognition through a structured diagnostic approach is crucial to guide appropriate management and prevent recurrence.

## Conclusions

Coronary embolism should be considered in patients presenting with myocardial infarction and non-obstructive coronary arteries, particularly in the presence of atrial fibrillation and rheumatic valvular heart disease. This case underscores the importance of a structured diagnostic approach to identify the underlying mechanism. Early recognition is crucial to guide appropriate management and reduce the risk of recurrent embolic events.

## References

[1] Tamis-Holland JE, Jneid H, Reynolds HR (2019). Contemporary diagnosis and management of patients with myocardial infarction in the absence of obstructive coronary artery disease: a scientific statement from the American Heart Association. Circulation.

[2] Pasupathy S, Air T, Dreyer RP, Tavella R, Beltrame JF (2015). Systematic review of patients presenting with suspected myocardial infarction and nonobstructive coronary arteries. Circulation.

[3] Shibata T, Kawakami S, Noguchi T (2015). Prevalence, clinical features, and prognosis of acute myocardial infarction attributable to coronary artery embolism. Circulation.

[4] Otto CM, Nishimura RA, Bonow RO (2021). 2020 ACC/AHA guideline for the management of patients with valvular heart disease: a report of the American College of Cardiology/American Heart Association Joint Committee on clinical practice guidelines. Circulation.

[5] January CT, Wann LS, Calkins H (2019). 2019 AHA/ACC/HRS focused update of the 2014 AHA/ACC/HRS guideline for the management of patients with atrial fibrillation: a report of the American College of Cardiology/American Heart Association Task Force on clinical practice guidelines and the Heart Rhythm Society in collaboration with the Society of Thoracic Surgeons. Circulation.

